# Sinomenine Ameliorated Microglial Activation and Neuropathic Pain After Chronic Constriction Injury Via TGF‐β1/ALK5/Smad3 Signalling Pathway

**DOI:** 10.1111/jcmm.70214

**Published:** 2024-11-25

**Authors:** Ling Ling, Min Luo, Haolin Yin, Yunyun Tian, Tao Wang, Bangjian Zhang, Li Yin, Yuehui Zhang, Jiang Bian

**Affiliations:** ^1^ Department of Anesthesiology Panzhihua Central Hospital Panzhihua Sichuan China; ^2^ The Third Affiliated Hospital of Zunyi Medical University The First People's Hospital of Zunyi Zunyi Guizhou China; ^3^ Department of Anesthesiology School of Clinic Medicine, Tsinghua University Beijing China; ^4^ Scientific Research and Discipline Construction Office Panzhihua Central Hospital Panzhihua Sichuan China; ^5^ Department of Anesthesiology, School of Clinic Medicine North Sichuan Medical University Nanchong Sichuan China; ^6^ Department of Neurology Panzhihua Central Hospital Panzhihua Sichuan China

**Keywords:** activin receptor–like receptor 5, microglia, neuropathic pain, sinomenine, Smad3, transforming growth factor‐β1

## Abstract

Sinomenine (SIN), a bioactive isoquinoline alkaloid extracted from the roots and stems of *Sinomenium acutum*, is efficacious against various chronic pain conditions. Inhibition of microglial activation at the spinal level contributes to the analgesic effects of SIN. Microglial activation in the spinal dorsal horn is key to sensitising neuropathic pain. Consequently, this study aimed to investigate whether the antinociceptive effects of SIN in neuropathic pain are induced through microglial inhibition and the underlying mechanisms. In this study, we observed that SIN alleviated chronic constriction injury (CCI)–induced pain hypersensitivity, spinal microglial activation and neuroinflammation. Consistently, SIN evoked the upregulation of transforming growth factor‐beta1 (TGF‐β1) and phosphorylated Smad3 in the L4–6 ipsilateral spinal dorsal horn of CCI mice. Intrathecal injection of TGF‐β1 siRNA and an activin receptor‐like receptor (ALK5) inhibitor reversed SIN's antinociceptive and antimicroglial effects on CCI mice. Moreover, targeting Smad3 in vitro with siRNA dampened the inhibitory effect of TGF‐β1 on lipopolysaccharide‐induced microglial activation. Finally, targeting Smad3 abrogated SIN‐induced pain relief and microglial inhibition in CCI mice. These findings indicate that the TGF‐β1/ALK5/Smad3 axis plays a key role in the antinociceptive effects of SIN on neuropathic pain, indicating its suppressive ability on microglia.

## 1 Introduction

Neuropathic pain is chronic pain caused by lesions or diseases of the somatosensory nervous system [1]. Spontaneous pain, hyperalgesia and allodynia are the typical symptoms associated with neuropathic pain. It is estimated that up to 10% of the general population suffers from neuropathic pain [2]. Clinical pharmacotherapies for neuropathic pain are inadequate for pain relief and usually cause unwanted adverse drug reactions, including fatigue, dizziness, nausea and anorexia [3]. Adverse reactions can significantly decrease compliance, even discontinuing before sufficient efficacy is achieved. Consequently, there is an urgent need for better treatment options.

Recent research has yielded a more comprehensive understanding of neuropathic pain pathogenesis. Neural plasticity, which refers to the functional and structural alterations in the peripheral nervous system (peripheral sensitisation), primarily in the dorsal root ganglion, spinal dorsal horn and supra‐spinal area (central sensitisation), is core to the occurrence and development of neuropathic pain [4]. If peripheral sensitisation increases pain sensitivity, central sensitisation eventually causes neuropathic pain by amplifying pain transmission and reducing the nociceptive threshold [5]. Regarding neuropathic pain, the current research emphasises exploring the mechanisms of central sensitisation. Several aberrant events happen in the spinal dorsal horn after peripheral nerve injury, especially the overactivation of microglia, contributing to the occurrence and maintenance of central sensitisation [6]. Neuroinflammation caused by microglial overactivation significantly enhances the excitability of secondary sensory neurons [7]. Therefore, overactivation of glial cells in the spinal dorsal horn is a potent promoter of neuropathic pain [8, 9].

Sinomenine (SIN) is a bioactive isoquinoline alkaloid extracted from the medicinal plant *Sinomenium acutum* [10]. SIN has been widely used to treat multiple pain conditions due to its anti‐inflammatory effects [11]. Increasing evidence proves that the therapeutic effects of SIN on chronic pain are achieved mainly via modulating immunocytes, inflammatory mediators and inflammation‐related signalling pathways [12, 13]. Mechanistic studies have revealed that inhibiting microglial‐induced neuroinflammation is a common mechanism for the neuroprotective effect of SIN [14, 15]. Recent evidence has exhibited that SIN ameliorated hyperalgesia and microglial activation in rodent models of neuropathic pain [12, 16]. However, the exact mechanism by which SIN‐induced analgesia regulates microglial activation remains largely unknown.

Many transforming growth factor‐beta (TGF‐β) family members are important modulators of pain processing [17]. TGF‐β1, a primary member of the TGF‐β family, is abundantly expressed in the central nervous system and exhibits an antinociceptive effect on neuropathic pain conditions, probably by inhibiting microglia‐induced neuroinflammation [18, 19]. The TGF‐β family initiates intracellular signalling by binding to type I and type II serine/threonine kinase receptors [20]. Activin receptor‐like receptor (ALK5) is a specific type I receptor of TGF‐β1 that drives phosphorylation of downstream Smad3 proteins [21]. TGF‐β/ALK5/Smad3 has been demonstrated to be a key signalling pathway to inhibit microglial activation in several neuropathological diseases [22, 23]. Studies have demonstrated that TGF‐β/Smad3 signalling has SIN‐related anti‐inflammatory effects in acute liver injury and asthma [24, 25]. However, whether the TGF‐β1/ALK5/Smad3 pathway is involved in ameliorating SIN in neuropathic pain remains unknown. Therefore, this study aimed to investigate the effects of SIN on chronic constriction injury–induced neuropathic pain and the role of TGF‐β1/ALK5/Smad3 in SIN's effects on neuropathic pain.

## 2 Materials and Methods

### 2.1 Mice

Male C57/BL6J mice (20–25 g, Charles River, China) used in the experiment were bred and maintained in ventilated cages, with 3–5 mice per cage. The mice were given free access to food and water and housed in a room with controlled temperature (22°C–25°C) and humidity (40%), with high‐efficiency particulate air filter (HEPA)‐filtered air supplied. The room was set to a 12‐h light/12‐h dark cycle. All experimental procedures were approved by the Institutional Animal Care and Use Committee of the North Sichuan Medical University (20220809‐005).

### 2.2 Experimental Design

To examine whether the chronic constriction injury (CCI) model could mimic neuropathic pain in mice, 12 mice were randomly divided into the sham group (mice with sham surgery) and CCI group (mice with chronic constriction of the sciatic nerve trifurcation), with six mice in each group. The pain behavioural tests were performed at baseline (1 day before CCI surgery) and 3, 7, 14 and 21 days after CCI surgery.

To investigate the analgesic effects of SIN on neuropathic pain, 60 mice were randomly divided into five groups: sham group (sham mice with intraperitoneal administration of vehicle); CCI group (CCI mice with intraperitoneal administration of vehicle); SIN‐5 mg/kg group (CCI mice with intraperitoneal administration of 5 mg/kg SIN); SIN‐10 mg/kg group (CCI mice with intraperitoneal administration of 10 mg/kg SIN) and SIN‐20 mg/kg group (CCI mice with intraperitoneal administration of 20 mg/kg SIN), with 12 mice in each group. The SIN administration began 15 days after CCI surgery and persisted for 21 days after CCI surgery. The pain behavioural test was performed 1 h before injection and 1, 2 and 4 h after injection to evaluate the effect of a single administration of SIN. The pain behavioural test was performed 1 day before injection, 3, 5 and 7 days after injection and 1 h before SIN injection to evaluate the effect of repeated administration of SIN. The spinal cord samples were harvested 21 days after CCI surgery (7 days of repeated SIN administration) to investigate whether SIN could affect the activation of microglia and the expression of proinflammatory cytokines, TGF‐β1 and phosphorylated Smad3.

To explore the spinal role of TGF‐β1 in the effects of SIN on CCI mice, TGF‐β1 small interfering RNA (siRNA) was used to decrease spinal TGF‐β1 expression. Initially, 18 mice were randomly divided into the vehicle group (mice with intrathecal injection of the vehicle), negative control (NC) siRNA group (mice with intrathecal injection of the NC siRNA) and TGF‐β1 siRNA group (mice with intrathecal injection of the TGF‐β1 siRNA), with six mice in each group. After 48 h of intrathecal injection of NC siRNA, nociceptive threshold and open‐field tests (OFT) were performed to investigate the side effects of intrathecal siRNA or reagents. The TGF‐β1 mRNA and protein expressions were detected in the L4–6 spinal dorsal horn to validate the knockdown effect of TGF‐β1 siRNA. Subsequently, 48 mice were divided into a sham group (sham mice with intrathecal injection of NC siRNA and intraperitoneal administration of vehicle), CCI group (CCI mice with intrathecal injection of NC siRNA and intraperitoneal administration of vehicle), SIN group (CCI mice with intrathecal injection of NC siRNA and intraperitoneal administration of SIN) and SIN+TGF‐β1 siRNA group (CCI mice with intrathecal injection of TGF‐β1 siRNA and intraperitoneal administration of SIN), with 12 mice in each group. The pain behavioural test was performed 1 day before the SIN injection and 3, 5 and 7 days after the SIN injection. Spinal cord samples were harvested after the last behavioural test to detect microglial activation and the expression of TGF‐β1 and phosphorylated Smad3.

To determine the potential receptors involved in the effects of SIN on CCI mice, 60 mice were randomly divided into a sham group (sham mice with intraperitoneal and intrathecal administration of the vehicle), CCI group (CCI mice with intraperitoneal and intrathecal administration of the vehicle), SIN group (CCI mice with intraperitoneal administration of SIN and intrathecal administration of the vehicle), SIN+ML347 group (CCI mice with intraperitoneal administration of SIN and intrathecal administration of ML347) and SIN+SB‐505124 group (CCI mice with intraperitoneal administration of SIN and intrathecal administration of SIN+SB‐505124), with 12 mice in each group. The pain behavioural test was performed 1 day before the SIN injection and 3, 5 and 7 days after the SIN injection. Spinal cord samples were harvested after the last behavioural test to detect microglial activation and the expression of TGF‐β1 and phosphorylated Smad3.

To examine the role of Smad3 in the effects of SIN on CCI mice, 48 mice were divided into a sham group (sham mice with intraperitoneal and intrathecal administration of the vehicle), CCI group (CCI mice with intraperitoneal and intrathecal administration of the vehicle), SIN group (CCI mice with intraperitoneal administration of SIN and intrathecal administration of the vehicle) and SIN+SIS3 group (CCI mice with intraperitoneal administration of SIN and intrathecal administration of SIS3), with 12 mice in each group. The pain behavioural test was performed 1 day before the SIN injection and 3, 5 and 7 days after the SIN injection. Spinal cord samples were harvested after the last behavioural test to detect microglial activation and expression of TGF‐β1 and phosphorylated Smad3.

### 2.3 CCI Model of Neuropathic Pain

CCI is a widely used neuropathic pain model in rodents because it can trigger persistent neuropathological responses, including marked glial activation and robust neuronal hypersensitivity in the peripheral and central nervous systems, inducing the sensitisation of neuropathic pain [26]. The CCI model was established as described by Bennet and Xie [27]. Briefly, the mice were anaesthetised with 2%–4% isoflurane, and a 1–2 cm incision was made at the mid‐thigh level of the right hind limb. The trifurcation of the sciatic nerve near the thigh region was freed from the adhering tissue, and three silk ligatures were gently tied around the trifurcation of the sciatic nerve, with approximately 1 mm between ligatures. For the sham surgery mice, the trifurcation of the sciatic nerve was exposed to the same site of the right thigh without ligating the nerve.

### 2.4 Behavioural Tests

Mechanical allodynia was assessed by detecting the paw withdrawal threshold (PWT). Briefly, the mice were acclimated for 30 min in Perspex chambers (10 × 5 × 5 cm^3^) placed on elevated wire mesh stands. Von Frey filament (0.02–2 g) was applied to the plantar surface of the hind paw, slightly bending the filaments for 3 s. A stronger filament was used if the hind paw did not lift within 3 s. Subsequently, a weaker filament was used if the paw was lifted after the filament stimuli. The stimulus force that elicited more than three paw withdrawals in the five tests was the PWT.

Thermal hyperalgesia was assessed by detecting thermal withdrawal latency (TWL). The mice were acclimated for 30 min in Perspex chambers (10 × 5 × 5 cm^3^) placed on a plexiglass floor. A thermal radiation source (Yuyan Instruments Company, Shanghai, China) was set at 52°C ± 0.5°C and focused on the centre area of the plantar surface of the hind paw three times, with 5‐min intervals between each stimulation. Thermal latency time was determined as the duration from heat exposure to hind paw withdrawal. The cutoff time for the thermal withdrawal test was 15 s. The TWL was determined by calculating the average withdrawal latency of three separate trials.

The motor function was assessed using OFT. Briefly, mice were placed in an open‐field box (45 × 45 × 30 cm^3^) using a high‐resolution video camera. Each mouse was allowed to explore for 5 min during the OFT. The average speed and total distance during exploration were calculated using a video tracking system (ANY‐maze V6.14; Stoelting, USA).

### 2.5 In Vivo siRNA Transfection

The siRNA targeting TGF‐β1 and NC were generated by OBIO Technology Co. Ltd. (Shanghai, China). For in vivo siRNA transfection, siRNAs were diluted in a mixture containing RNase‐free water, branched polyethyleneimine (4052836, Thermo Fisher Scientific, USA) and 5% glucose to a concentration of 2 μg/μL. Briefly, 2 μL of siRNA solution was injected intrathecally into CCI mice 1 and 7 days after CCI surgery. Transfection efficiency was validated by measuring TGF‐β1 protein and mRNA expression in the spinal dorsal horn. The siRNA sequences were as follows: TGF‐β1 siRNA‐1: 5′‐CCAGCCGCGGGACUCUCCACCUGCA‐3′; TGF‐β1 siRNA‐2: 5′‐GAGGAAAAAAGUUUUGAGACU‐3′; NC siRNA: 5′‐CCAGCGCGGCACUCUACCCUCGGCA‐3′.

### 2.6 BV2 Cell Culture and siRNA Transfection

The BV2 microglial cells (SNL‐155, SUNNCELL, Wuhan, China) were cultured in Dulbecco's modified Eagle's medium with 10% foetal bovine serum at 37°C and 5% CO_2_. Smad3 or NC siRNA was transfected into cultured BV2 cells (1 × 10^5^) using lipofectamine RNAi MAX (Thermo Fisher Scientific. USA). The siRNA sequences were as follows: Smad3 siRNA: 5′‐ACGCAGAACGUGAACACCAAGUGCA‐3′; NC siRNA: 5′‐ACGCAAGAGUGCACAGAACUACGCA‐3′. After culturing for 24 h, the medium containing the siRNAs and transfection reagents was replaced with a fresh medium for subsequent experiments. Then, lipopolysaccharide (LPS, HY‐D1056; MedChemExpress, USA) was added to the medium at a concentration of 100 ng/mL to activate BV2 cells. The stock TGF‐β1 solution (1 μg/mL) was prepared in dimethyl sulfoxide (DMSO) and added to the medium at a concentration of 10 ng/mL to examine the effects of TGF‐β1. The TGF‐β1 was present during LPS treatment. After culturing for 12 h, the cells were harvested to validate transfection efficiency and other detection methods.

### 2.7 Intrathecal Injection

The intrathecal injection was performed using the lumbar puncture method, as previously described [28]. The mice were prone, and the area around the L5–6 spine level was exposed and disinfected. A 20‐gauge needle connected to a 10‐μL Hamilton microsyringe was inserted into the subarachnoid space through the L5–6 spinous space. A quick tail flick was considered a sign of dura penetration of the needle.

### 2.8 Drug Administration

SIN (115‐53‐7; Solarbio, Beijing, China) was dissolved in 10% DMSO (HY‐Y0320; MedChemExpress, NJ, USA) and intraperitoneally administered to CCI mice in a dose‐dependent manner to examine the analgesic effects of SIN in mice. The ALK1 selective inhibitor ML347 (100 ng, HY‐12274; MedChemExpress, NJ, USA) or ALK5 selective inhibitor ALK5 inhibitor SB‐505124 (100 ng, HY‐13521; MedChemExpress, NJ, USA) was dissolved in 2 μL of 10% DMSO and intrathecally administered to CCI mice following treatment with SIN to determine which receptor was involved in the effects of SIN on CCI mice. The selective Smad3 inhibitor, SIS3 (100 ng, HY‐13013; MedChemExpress, NJ, USA), was intrathecally administered to CCI mice following SIN treatment to confirm that Smad3 phosphorylation was responsible for SIN‐induced microglial inhibition.

### 2.9 Immunofluorescence

The mice were narcotised with 2% isoflurane and intracardially perfused with 0.01 M phosphate buffer solution (PBS) to perform immunofluorescence staining of spinal cord samples. Following perfusion with 4% paraformaldehyde, L4–6 spinal cord segments were obtained by retro‐tracing the sciatic nerve trunk and fixed with 4% paraformaldehyde for 6 h. The fixed spinal cord samples were transferred to a 30% sucrose solution and dehydrated for 12 h. Transverse spinal cord sections (20 μm) were obtained using a microtome (CM1900; Leica, Wetzlar, Germany). After sample preparation, L4–6 spinal cord sections were permeabilised with 0.3% Triton X‐100 for 15 min and blocked with 10% goat serum for 30 min at room temperature. Subsequently, the sections were incubated overnight with the primary antibodies at 4°C. The primary antibodies were as follows: mouse anti‐Iba‐1 antibody (1:100, ab283319, Abcam), rabbit anti‐TGF‐β1 antibody (1:100, ab314095, Abcam) and rabbit antiphosphorylated Smad3 (p‐Smad3) antibody (1:200, ab52903, Abcam). The next day, sections were rinsed with 0.01 M PBS and incubated with secondary antibodies at room temperature for 2 h. The secondary antibodies were as follows: goat antimouse IgG H&L (Alexa Fluor 488, 1:500, ab150113, Abcam), goat antirabbit IgG H&L (Alexa Fluor 555, 1:500, ab150170, Abcam) and goat antimouse IgG H&L (Alexa Fluor 555, 1:500, ab150114, Abcam). The sections were washed with 0.01 M PBS and stained with 4,6‐diamino‐2‐phenyl indole (DAPI) at room temperature for 5 min. Fluorescence images of the sections were captured using a confocal microscope.

For in vitro immunofluorescence staining, BV2 microglial cells were collected on glass slides and fixed with 4% paraformaldehyde for 15 min. Fixed microglia were permeabilised with 0.3% Triton X‐100 for 10 min. Nonspecific antigens were blocked by incubation with 10% donkey serum for 30 min. Microglia were incubated with mouse anti‐Iba‐1 antibody (1:100, ab283319, Abcam) at 4°C for 2 h. The cells were washed three times in 0.01 M PBS and incubated with donkey antimouse IgG H&L (Alexa Fluor 488, 1:500, ab150113, Abcam) secondary antibody for 2 h at room temperature. The cells were washed three times with 0.01 M PBS and stained with DAPI solution. Fluorescence images of the cells were acquired using a confocal microscope.

The fluorescence in the sections was quantified at 200× magnification using ImageJ software. Every third spinal cord section (100 μm apart) was averaged per mouse, with six mice in each group. As described previously, the integrated optical density of the target proteins was estimated by the total pixel value of the target protein/total unfiltered pixel value of the region of interest (ROI) [29]. The area of p‐Smad3 colocalisation with Iba‐1 was calculated to represent p‐Smad3 expression in microglia. The cultured cells were counted at 400× magnification using the ImageJ software. Four fields were averaged per well with six replicates per group.

### 2.10 Western Blot (WB) Analysis

Mice were anaesthetised with 2% isoflurane and intracardially perfused to obtain L4–6 spinal cord tissue. The L4–6 spinal cord segments were gently divided into two hemi‐sections along the median in the vertical direction. Then, Ipsilateral and contralateral hemi‐sections of the CCI mice were separated into the dorsal and ventral horns via the boundary of the substantia gelatinosa using a microscope. Dorsal horn tissues were prepared for the WB analysis. The cultured BV2 cells were separated from the medium. Total protein was extracted from the samples using radio immunoprecipitation assay (RIPA) lysis buffer (P0038, Beyotime, China). The total protein concentration was determined using a bicinchoninic acid (BCA) kit (P0009, Beyotime, China). Denatured proteins were obtained by heating the samples at 99°C for 5 min. Subsequently, the proteins were separated using 10% SDS–PAGE and transferred onto polyvinylidene fluoride membranes. The membranes were immersed in 5% bovine serum albumin for 1 h at room temperature to block nonspecific reaction antigens, followed by overnight incubation with primary antibodies at 4°C. The primary antibodies were as follows: mouse anti‐Iba‐1 antibody (1:1000, ab283319, Abcam), rabbit anti‐TGF‐β1 antibody (1:1000, ab314095, Abcam), rabbit antiphosphorylated Smad3 (p‐Smad3) antibody (1:2000, ab52903, Abcam), rabbit anti‐TNF‐α antibody (1:1000 ab183218, Abcam), rabbit anti‐IFN‐γ antibody (1:1000, HY‐P81111, medchemexpress) and rabbit anti‐GAPDH antibody (1:5000, AF1186, Beyotime, China). Then, the membranes were washed three times with Tris‐buffered saline with Tween‐20 (TBST), followed by incubation with horseradish peroxidase–conjugated goat anti‐rabbit antibody (1:3000, 31460, Invitrogen, USA) or horseradish peroxidase–conjugated goat antimouse antibody (1:3000, 31430, Invitrogen, USA) for 2 h at room temperature. Protein bands were detected using enhanced chemiluminescence reagents. Immunoblotting images were acquired using an LI‐COR/Odyssey infrared image system (Bio‐Rad, ChemiDoc XRS+, USA). Immunoblotting density was calculated using the ImageJ software. The expression levels of target proteins were evaluated by normalising the band density to GAPDH.

### 2.11 Quantitative Real‐Time Polymerase Chain Reaction (qRT‐PCR)

The spinal cord tissues and cultured cells were collected using the same methods as WB. Total RNA from L4–6 spinal cords was extracted using an RNA extraction reagent (R401‐01; Vazyme Biotechnology, Nanjing, China) according to the manufacturer's instructions. Total RNA concentrations in the test samples were measured using a NanoDrop 2000 spectrophotometer (Thermo Scientific). Total RNA was reverse‐transcribed into cDNA using PrimeScript RT Master Mix (RR036A, Takara, Japan). The qRT‐PCR was performed using an RT‐PCR system (Roche, Switzerland) under standard conditions. The expression levels of the target genes were analysed using the 2^−ΔΔCt^ method, with normalisation to the GAPDH internal control gene. The primers were as follows: TGF‐β1 (Accession: NM_011577): sense: 5′‐CTGCTGACCCCCACTGATAC‐3′; antisense: 5′‐GGGGCTGATCCCGTTGATT‐3′; Smad3 (Accession: NM_016769): sense: 5′ACACTAACTTCCCTGCTGGC‐3′, antisense: 5′AGAGGTTTGGAGAACCTGCG‐3′.

GAPDH (Accession: NM_001289726): sense: 5′‐AGGTCGGTGTGAACGGATTTG‐3′ antisense: 5′‐TGTAGACCATGTAGTTGAGGTCA‐3′.

### 2.12 Cell Counting Kit (CCK‐8) Assay

The viability of cultured BV2 cells was determined using CCK‐8 (C008, Beyotime Biotechnology, Shanghai, China). After siRNA and LPS treatment, BV2 microglial cell intensities were adjusted to 10,000 cells/well in 96‐well plates. Finally, the primary medium in each well was replaced with a fresh medium containing 10% CCK‐8. After incubation with CCK‐8 for 4 h, cell viability was measured by detecting the absorbance at 450 nm.

### 2.13 Statistical Analysis

The data were processed using the Statistical Package for the Social Sciences software (version 17.0; SPSS Inc., Chicago, IL, USA). Normality and equal variance were determined using the Shapiro–Wilk normality and Brown–Forsythe tests. PWT and TWL between multiple groups at different time points were compared using repeated measures analysis of variance (ANOVA), followed by Bonferroni's post hoc test. WB and immunofluorescence staining data were compared using one‐way ANOVA, followed by Bonferroni's post hoc test or independent sample *t*‐test. Nonparametric data were evaluated using the Kruskal–Wallis test, followed by Dunn's test. The level of statistical significance was set at *p* < 0.05.

## 3 Results

### 3.1 SIN Alleviates Pain Sensitisation in CCI Mice

**FIGURE 1:**
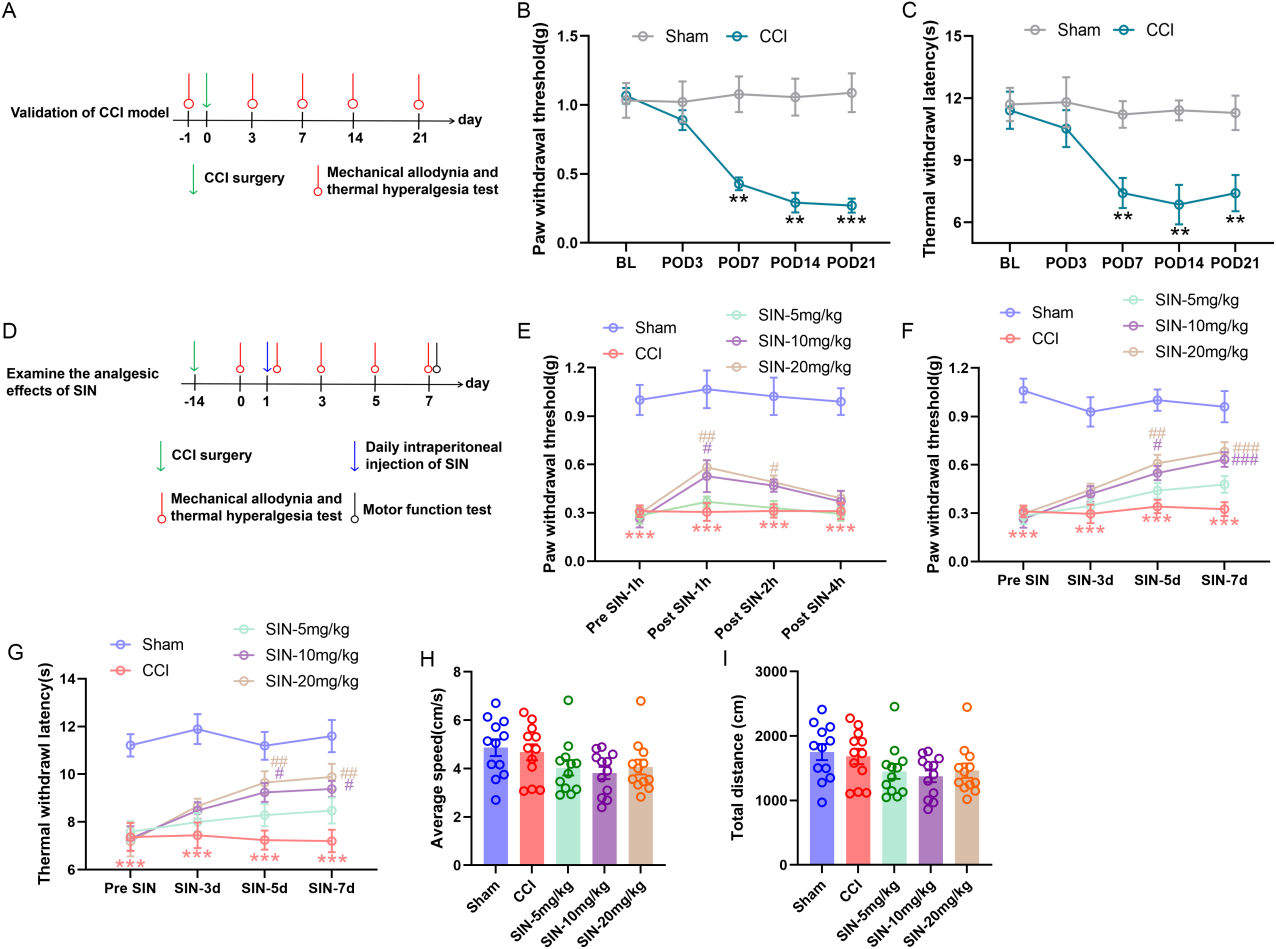
SIN alleviated CCI‐induced mechanical allodynia and thermal hyperalgesia. (A) The schematic for validating the CCI model. (B and C) PWT and TWL of sham and CCI mice were measured at baseline and POD 3, 7, 14 and 21, data are presented as mean ± standard error of mean (SEM); CCI group versus Sham group **p < 0.01, ***p < 0.001, *n* = 6. (D) The schematic for examining the analgesic effects of the SIN. (E) The time course of PWT in sham or CCI mice with a single administration of different doses of SIN or vehicle. (F and G) The time course of PWT and TWL in sham or CCI mice with repetitive administration of different doses of SIN or vehicle. (H and I) The average speed and total distance in the open field were measured after 7 days of SIN treatment. Data are presented as the mean ± SEM; CCI group versus sham group ***p < 0.001; SIN‐10 mg/kg and SIN‐20 mg/kg groups versus CCI group ^#^ *p* < 0.05, ^##^ *p* < 0.01, ^###^ *p* < 0.001, *n* = 12.

The CCI model was established to explore the antinociceptive effect of SIN on neuropathic pain in mice, and pain behaviours were measured on preoperative day 1 and postoperative days (POD) 3, 7, 14 and 21. The behavioural test results revealed that pain hypersensitivity developed in CCI mice on POD 7 and was persistent over 2 weeks (Figure 1A–C), characterised by mechanical allodynia and heat hyperalgesia compared to the sham mice (PWT: repeated measure ANOVA *F*_1,10_ = 29.250, *p* < 0.001; TWL: repeated measure ANOVA *F*_1,10_ = 23.784, *p* = 0.001). Next, we assessed the effects of SIN on CCI‐induced pain by intraperitoneal injection of different doses of SIN into CCI mice starting on POD 14 (Figure 1D). Single administration of SIN dose dependently ameliorated CCI‐induced pain hypersensitivity. The analgesic effect continued for at least 2 h, with the most effective repression occurring 1 h after administration (PWT: repeated measure ANOVA *F*_4,55_ = 109.987, *p* < 0.001) (Figure 1E). To investigate the long‐term analgesic effect of SIN, the ipsilateral PWT and TWL were measured immediately before SIN injection on days 3, 5 and 7 of repetitive SIN treatment and day 1 before SIN treatment. According to behavioural test results, the cumulative analgesic effect of SIN started on day 5 of repetitive SIN treatment, characterised by the significant elevation of PWT and TWL values in SNI‐10 mg and SNI‐20 mg groups in comparison to the CCI group (Figure 1F,G). In addition, significant pain relief was maintained until day 7 of repetitive SIN treatment, without significant differences in mechanical allodynia and thermal hyperalgesia tests between day 5 and day 7 of SNI‐10 mg and SNI‐20 mg groups (PWT: repeated measure ANOVA *F*_4,55_ = 126.681, *p* < 0.001, TWL: repeated measure ANOVA *F*_4,55_ = 43.027, *p* < 0.001) (Figure 1F,G). To investigate whether the SIN treatment could affect the mouse's motor function, we measured the average speed and total distance of mice freely moving in an open field. As a result, repetitive administration of any concentration of SIN did not significantly affect the average speed and total distance in the open field, indicating no sedation or locomotor impairment (Figure 1H,I) (average speed: one‐way ANOVA *F*_4,55_ = 2.104, *p* = 0.0094, total distance: one‐way ANOVA *F*_4,55_ = 2.108, *p* = 0.092). These results indicated that SIN exerted an analgesic effect in neuropathic pain conditions.

### 3.2 SIN Inhibited Microglial Activation and Enhanced TGF‐β1 Expression and Smad3 Phosphorylation in the Spinal Dorsal Horn of Mice With CCI

**FIGURE 2:**
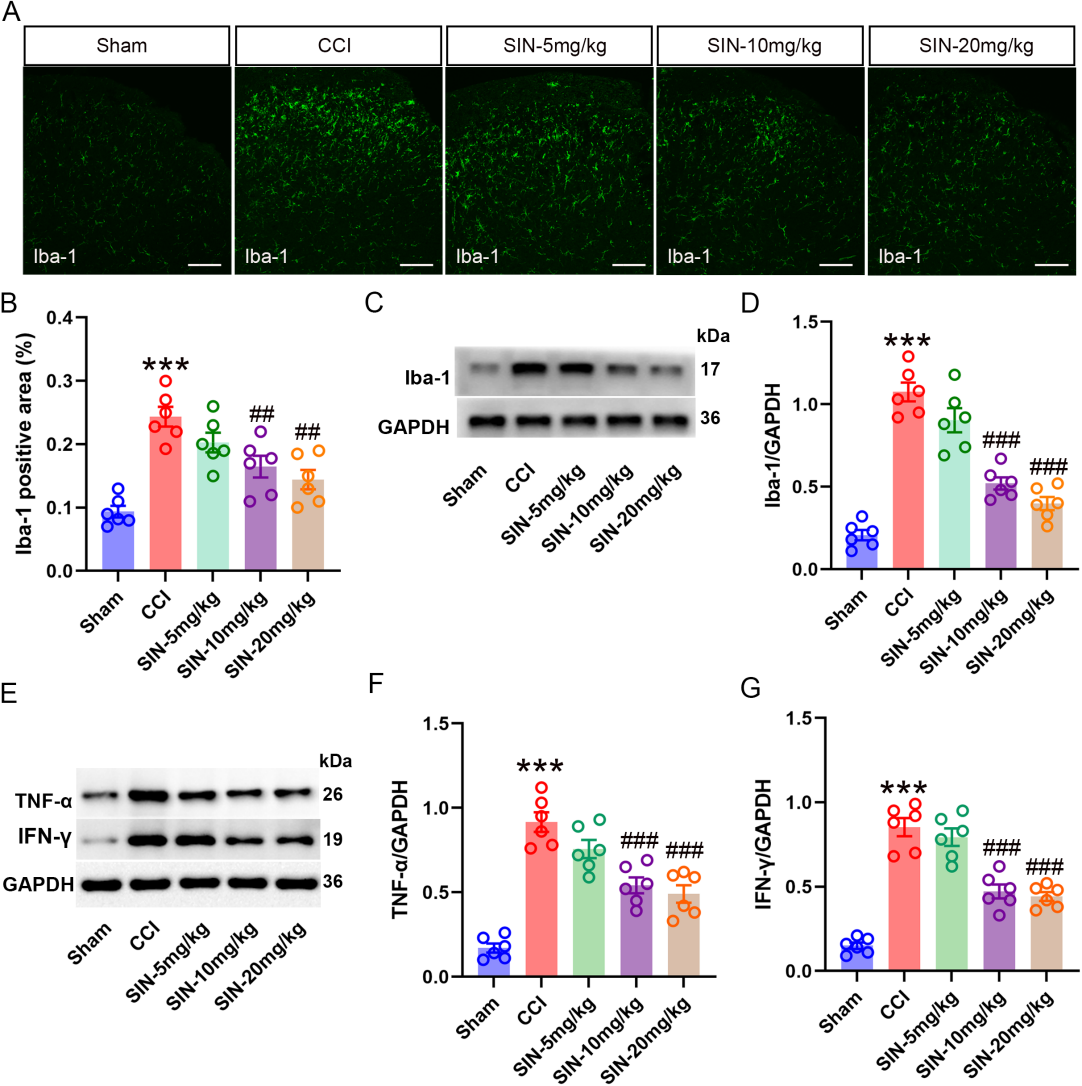
SIN inhibited microglial activation and neuroinflammation in the ipsilateral spinal dorsal horn of CCI mice. (A and B) Immunofluorescence staining indicated Iba‐1 expression in the ipsilateral spinal dorsal horn (scale bar = 100 μm). (C and D) WB analysis showed the Iba‐1 expression level in the ipsilateral spinal dorsal horn. (E–G) WB analysis showed the TNF‐α and IFN‐γ expression levels in the ipsilateral spinal dorsal horn. Data are presented as mean ± SEM; CCI group versus sham group ***p < 0.001; SIN‐10 mg/kg and SIN‐20 mg/kg groups versus CCI group ^##^ *p* < 0.01, ^###^ *p* < 0.001, *n* = 6.

**FIGURE 3:**
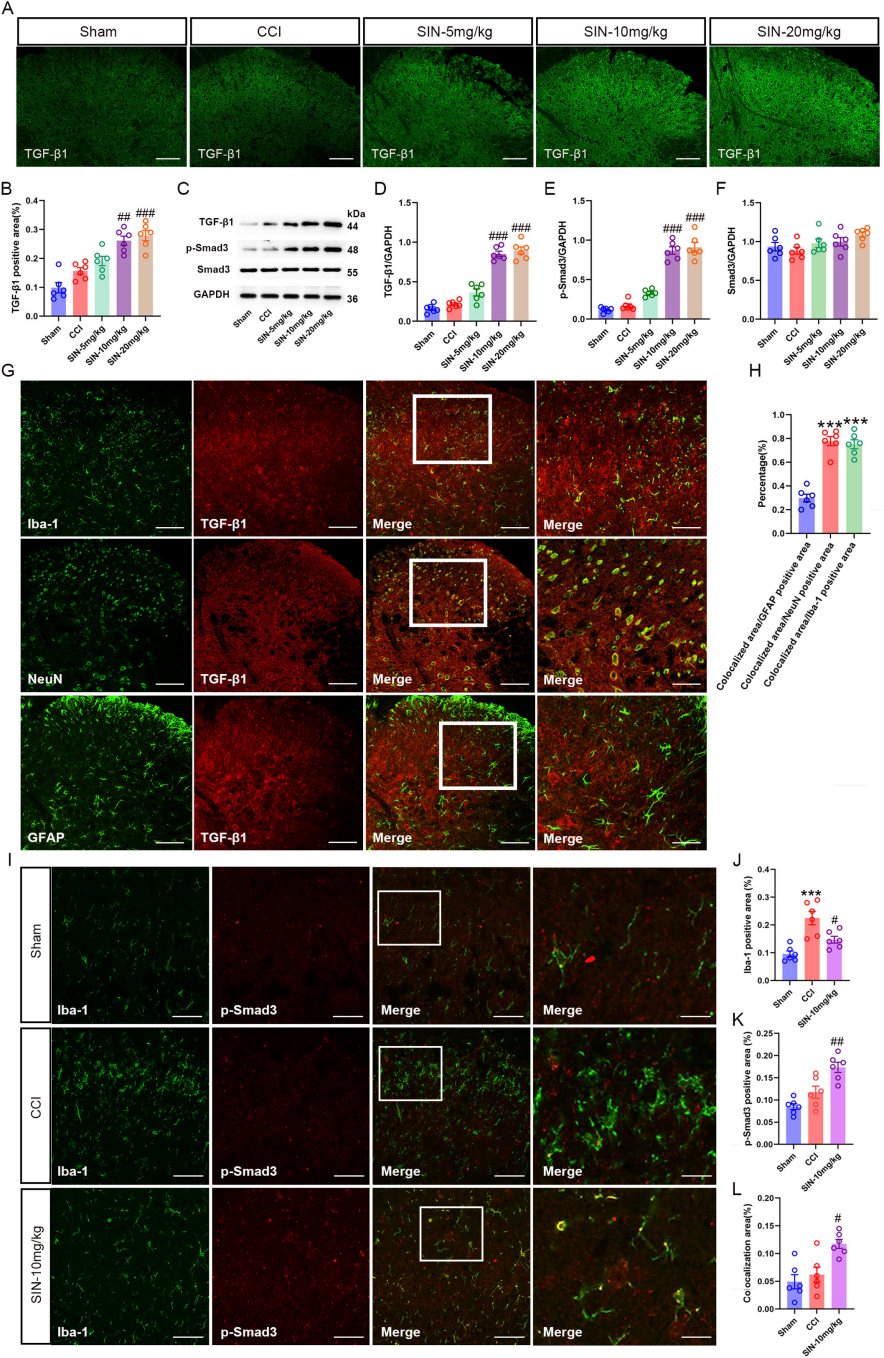
SIN enhanced TGF‐β1 and p‐Smad3 expression in the ipsilateral spinal dorsal horn of CCI mice. (A and B) Immunofluorescence staining revealed TGF‐β1 expression in the ipsilateral spinal dorsal horn (scale bar = 100 μm), (C–F) WB analysis showed the TGF‐β1, p‐Smad3 and p‐Smad3 expression levels in the ipsilateral spinal dorsal horn, data are presented as mean ± SEM; CCI group versus sham group ***p < 0.001; SIN‐10 mg/kg and SIN‐20 mg/kg groups versus CCI group ^##^ *p* < 0.01, ^###^ *p* < 0.001, *n* = 6. (G and H) Double immunofluorescence staining showed the coexpression of TGF‐β1 (red) with Iba‐1, NeuN, GFAP (green) in the ipsilateral spinal dorsal horn (scale bar = 100 μm), data are presented as mean ± SEM; Colocalised area/NeuN positive area and Colocalised area/Iba‐1 positive area versus Colocalised area/GFAP positive area ***p < 0.001, *n* = 6. (I–L) Double immunofluorescence staining showed the coexpression of Iba‐1 (green) with p‐Smad3 (red) in the ipsilateral spinal dorsal horn (scale bar = 50 μm), data are presented as mean ± SEM; CCI group versus sham group ***p < 0.001; SIN‐10 mg/kg group versus CCI group ^#^ *p* < 0.05, ^##^ *p* < 0.01, *n* = 6.

Microglial activation is a well‐established pathological alteration in neuropathic pain. The activated microglia released abundant inflammatory cytokines, causing hyperexcitation of neurons involved in pain transmission [30]. In this study, CCI facilitated the expression of Iba‐1 (microglial marker) in the ipsilateral spinal dorsal horn on POD 21 (Iba‐1 positive area: one‐way ANOVA *F*_4,25_ = 14.750, *p* < 0.001; Iba‐1/GAPDH: one‐way ANOVA *F*_4,25_ = 51.44, *p* < 0.001) (Figure 2A–D), indicating microglial activation. Meanwhile, TNF‐α and IFN‐γ upregulation was consistent with microglial activation (TNF‐α/GAPDH: one‐way ANOVA *F*_4,25_ = 33.88, *p* < 0.001; IFN‐γ/GAPDH: one‐way ANOVA *F*_4,25_ = 49.31, *p* < 0.001) (Figure 2E–G). SIN treatment dose dependently inhibited the upregulation of Iba‐1, TNF‐α and IFN‐γ in the ipsilateral spinal dorsal horn of CCI mice, indicating that microglial activation and neuroinflammation are involved in SIN‐induced analgesia. Following the 7 days of SIN treatment, we found that TGF‐β1 expression was significantly enhanced in the spinal dorsal horn of mice treated with 10 and 20 mg/kg of SIN than in CCI mice (TGF‐β1 positive area: one‐way ANOVA *F*_4,25_ = 21.80, *p* < 0.001; TGF‐β1/GAPDH: one‐way ANOVA *F*_4,25_ = 102.19, *p* < 0.001) (Figure 3A–D). To examine the expression pattern of the upregulated TGF‐β1, we performed the double‐staining immunofluorescence in the ipsilateral spinal dorsal horn of mice with 7 days of 10 mg/kg of SIN treatment to observe the colocalisation of the TGF‐β1 with NeuN (neuronal marker), GFAP (astrocytic marker) and Iba‐1 (astrocytic marker). As a result, the increased TGF‐β1 was prone to colocalise with NeuN and Iba‐1 in comparison to GFAP (one‐way ANOVA *F*_2,15_ = 54.10, *p* < 0.001) (Figure 3G,H), indicating that the SIN might accelerate the expression in neurons and microglia. Consistent with the upregulation of TGF‐β1, 10 and 20 mg/kg of SIN treatment also promoted the phosphorylation of Smad3, without influencing its expression (p‐Smad3/GAPDH: one‐way ANOVA *F*_4,25_ = 102.53, *p* < 0.001, Smad3/GAPDH: one‐way ANOVA *F*_4,25_ = 2.53, *p* = 0.66) (Figure 3C,E,F). Moreover, the upregulated phosphorylated‐Smad3 (p‐Smad3) was highly colocalised with the Iba‐1, and the colocalised area of mice treated with SIN was larger than CCI or sham mice (Iba‐1 positive area: one‐way ANOVA *F*_4,25_ = 14.764, *p* < 0.001; p‐Smad3 positive area: one‐way ANOVA *F*_4,25_ = 16.396, *p* < 0.001; colocalised area: one‐way ANOVA *F*_4,25_ = 9.532, *p* = 0.002) (Figure 3I–L). As Smad3 is an important intracellular mediator of TGF‐β1, its phosphorylation is regulated by TGF‐β1 [31]. Therefore, this increased colocalisation of p‐Smad3 with Iba‐1 could represent the activation of TGF‐β1/Smad3 signalling in microglia. Collectively, these results indicated that TGF‐β1/Smad3 signalling was closely associated with SIN‐induced microglial inhibition in CCI mice.

### 3.3 Knockdown of TGF‐β1 Expression Reversed SIN's Beneficial Effects of SIN in CCI Mice

**FIGURE 4:**
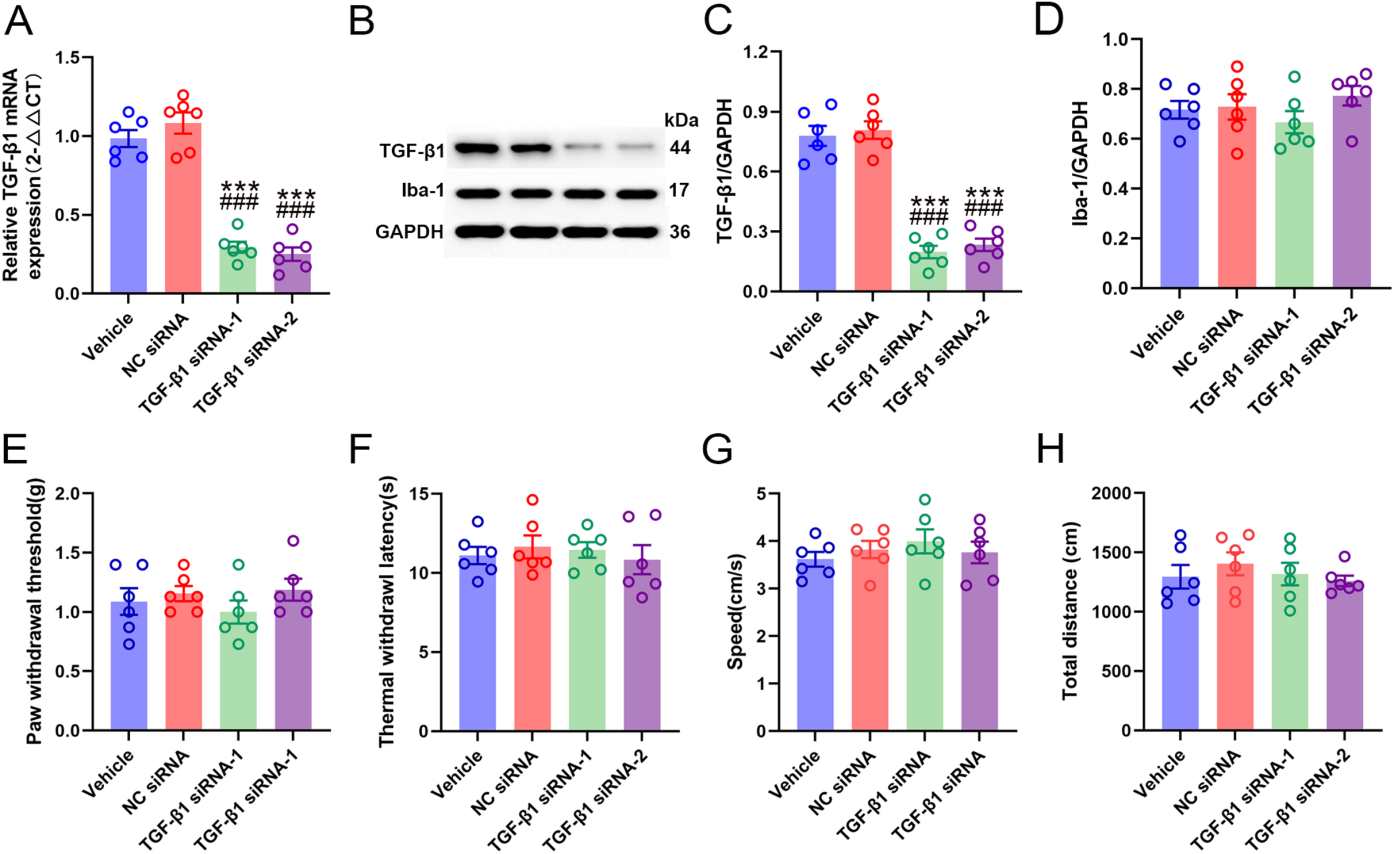
Intrathecal injection of TGF‐β1 siRNA decreased TGF‐β1 expression without conspicuous sensory and motor impairments. (A) qRT‐PCR analysis exhibited TGF‐β1 mRNA expression levels in the spinal dorsal horn of mice after intrathecal injection of vehicle, NC siRNA and TGF‐β1 siRNAs. (B–D) WB analysis displayed TGF‐β1 and Iba‐1 protein expression levels in the spinal dorsal horn of mice after intrathecal injection of vehicle, NC siRNA and TGF‐β1siRNAs. (E and F) PWT and TWL of mice after intrathecal injection of vehicle, NC siRNA and TGF‐β1 siRNAs. (G and H) Average speed and total distance in the OFT of mice after intrathecal injection of vehicle, NC siRNA and TGF‐β1 siRNAs. Data are presented as the mean ± SEM; TGF‐β1 siRNA‐1 and TGF‐β1 siRNA‐2 groups versus vehicle group ***p < 0.001; TGF‐β1 siRNA‐1 and TGF‐β1 siRNA‐2 groups versus NC siRNA group ^###^ *p* < 0.001, *n* = 6.

**FIGURE 5:**
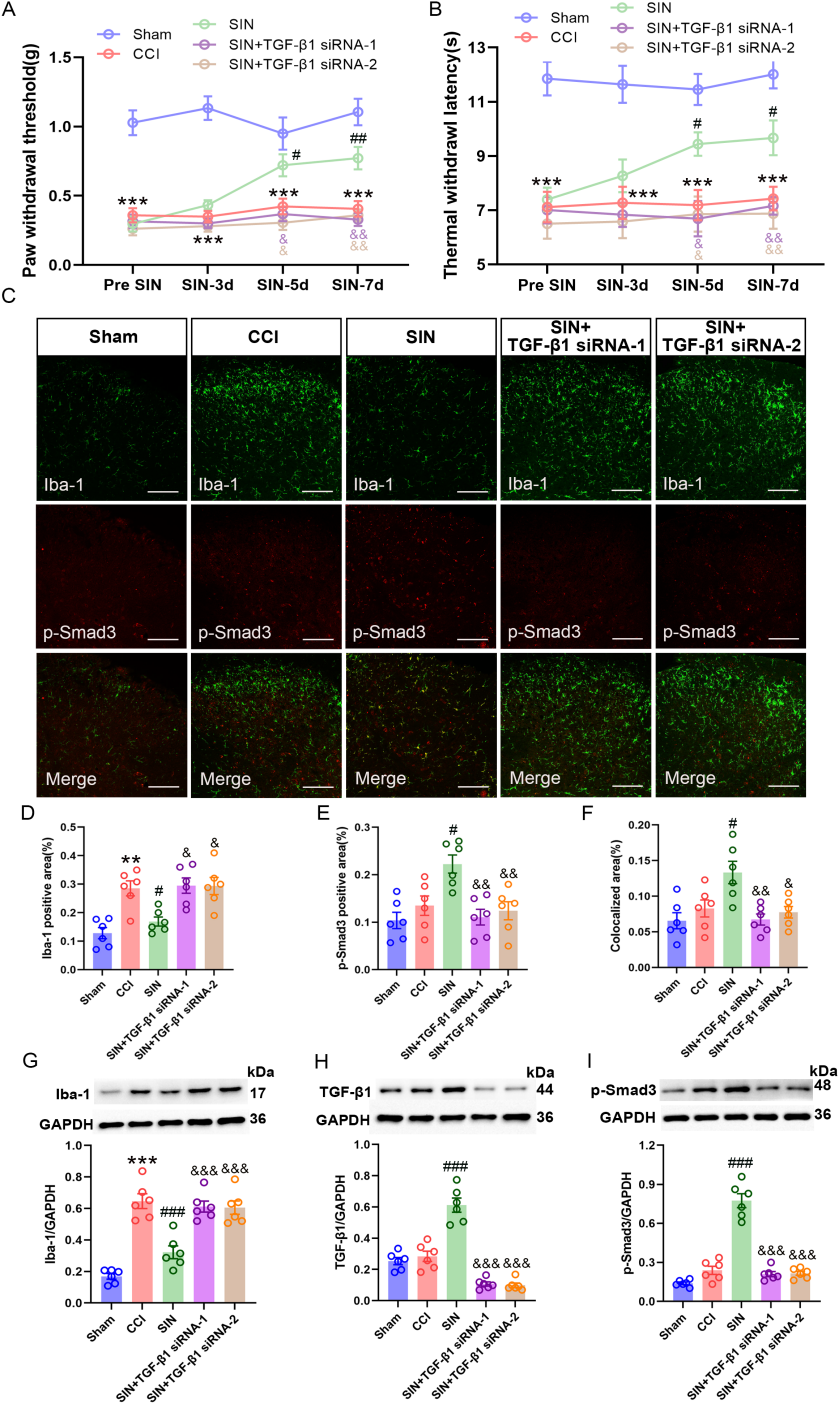
Intrathecal injection of TGF‐β1 siRNAs reversed SIN‐induced pain amelioration and microglial inhibition. (A and B) The time course of PWT and TWL in sham and CCI mice after TGF‐β1 siRNA delivery and SIN treatment, data are presented as mean ± SEM, Sham group versus CCI group ***p < 0.001; SIN group versus CCI group ^##^ *p* < 0.01, ^###^ *p* < 0.001; SIN+TGF‐β1 siRNA group versus SIN group ^&^ *p* < 0.05, ^&&^ *p* < 0.01, *n* = 12. (C–F) Double immunofluorescence staining indicated the expression of Iba‐1 (green) with p‐Smad3 (red) in the ipsilateral spinal dorsal horn of sham and CCI mice after TGF‐β1 siRNA delivery and SIN treatment (scale bar = 100 μm). (G–I) WB showed the Iba‐1, TGF‐β1 and p‐Smad3 expression levels in the ipsilateral spinal dorsal horn of sham and CCI mice after TGF‐β1 siRNA delivery and SIN treatment. Data are presented as mean ± SEM. Sham group versus CCI group **p < 0.001, ***p < 0.001; SIN group versus CCI group ^#^ *p* < 0.05, ^##^ *p* < 0.01, ^###^ *p* < 0.001; SIN+TGF‐β1 siRNA‐1 and SIN+TGF‐β1‐2 siRNA group versus SIN group ^&&^ *p* < 0.01, ^&&&^ *p* < 0.001, *n* = 6.

After observing that SIN induced potent TGF‐β1 expression in the spinal microglia, two TGF‐β1 siRNA sequences were intrathecally injected to examine whether TGF‐β1 was involved in SIN‐induced antiallodynia and antimicroglial activation by silencing its spinal expression. At first, the knockdown efficiency of TGF‐β1 siRNAs was validated in the normal mice, and the L4–6 spinal dorsal horns of the mice were harvested 48 h after the TGF‐β1 siRNAs intrathecal injection. Here, qRT‐PCR and WB results revealed that TGF‐β1 mRNA and protein expressions in the L4–6 spinal dorsal horns of normal mice with these two TGF‐β1 siRNAs intrathecal injection were decreased in comparison with those of normal mice with vehicle or NC siRNA intrathecal injection (TGF‐β1 mRNA: one‐way ANOVA *F*_3,20_ = 73.64, *p* < 0.001; TGF‐β1/GAPDH: one‐way ANOVA *F*_3,20_ = 70.31, *p* < 0.001) (Figure 4A–C), indicating the successful transfection of TGF‐β1 siRNAs. Moreover, the expression of Iba‐1 remained unchanged with the TGF‐β1 siRNAs intrathecal injection, suggesting that knockdown of spinal TGF‐β1 did not affect the activity of resting microglia in normal mice (Iba‐1/GAPDH: one‐way ANOVA *F*_3,20_ = 1.03, *p* = 0.401) (Figure 4B,D). Next, we explored whether the knockdown of spinal TGF‐β1 could cause unwanted effects on sensory or motor function. To investigate the sensory function, we detected the mouse's paw to mechanical and thermal stimuli after 48 h of TGF‐β1 siRNAs intrathecal injection. Following the sensory tests, the average speed and total distance travelled by the mice in an open field were detected to evaluate any influence on motor function of TGF‐β1 siRNAs intrathecal injection. As a result, no significant differences were found in PWT, TWL, average speed and total distance of mice receiving vehicle, TGF‐β1 siRNA‐1, TGF‐β1 siRNA‐2 or NC siRNA (PWT: one‐way ANOVA *F*_3,20_ = 0.80, *p* = 0.510; TWL: one‐way ANOVA *F*_3,20_ = 0.27, *p* = 0.844; average speed: one‐way ANOVA *F*_3,20_ = 0.56, *p* = 0.647; total distance: one‐way ANOVA *F*_3,20_ = 0.50, *p* = 0.687) (Figure 4E–H), indicating negligible side effects of TGF‐β siRNAs spinal transfection. TGF‐β1 siRNA‐1 and TGF‐β1 siRNA‐2 were intrathecally injected into CCI mice on POD 1 and 7 to explore the involvement of spinal TGF‐β1 in the beneficial effects of SIN on CCI mice. Notably, CCI evoked pain hyperalgesia and microglial activation. However, these CCI‐induced alterations were blocked following 7 days of SIN treatment (PWT: repeated measure ANOVA *F*_4,55_ = 99.71, *p* < 0.001; TWL: repeated measure ANOVA *F*_4,55_ = 40.59, *p* < 0.001; Iba‐1 positive area: one‐way ANOVA *F*_4,25_ = 10.74, *p* < 0.001; Iba‐1/GAPDH: one‐way ANOVA *F*_4,25_ = 31.59, *p* < 0.001) (Figure 5A–D,G). As expected, the SIN‐mediated beneficial effects on pain sensitisation and microglial activation disappeared without TGF‐β1 (TGF‐β1: one‐way ANOVA *F*_4,25_ = 53.71, *p* < 0.001) (Figure 5H). SIN‐induced upregulation of p‐Smad3 in microglia was also reversed in the absence of TGF‐β1 (p‐Smad3 positive area: one‐way ANOVA *F*_4,25_ = 6.75, *p* = 0.001; colocalised area: one‐way ANOVA *F*_4,25_ = 6.00, *p* = 0.002; p‐Smad3/GAPDH: one‐way ANOVA *F*_4,25_ = 72.47, *p* < 0.001) (Figure 5C,E,F,I). These results revealed the indispensable role of TGF‐β1 in SIN‐induced antiallodynia and microglial inhibition in CCI mice.

### 3.4 ALK5, Not ALK1, is Responsible for the Protective Effects of SIN

**FIGURE 6:**
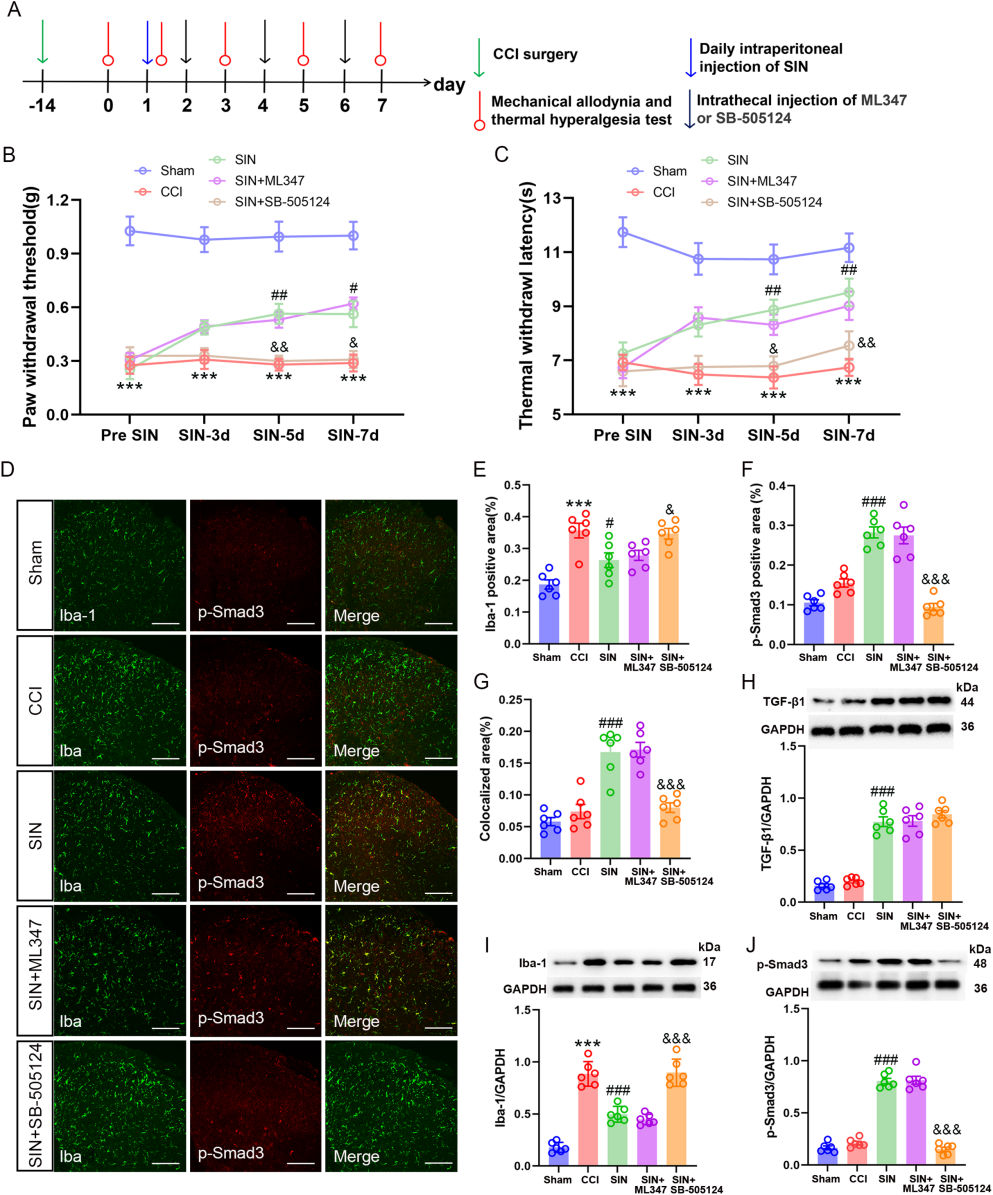
Intrathecal injection of an ALK5 inhibitor abrogated SIN‐induced pain amelioration and microglial inhibition. (A) Schematic for determining the binding receptors involved in the effects of SIN. (B and C) Time course of PWT and TWL in sham and CCI mice after SIN treatment and the inhibitors intrathecal delivery, data are presented as mean ± SEM, Sham group versus CCI group ***p < 0.001; SIN group versus CCI group ^#^ *p* < 0.05, ^##^ *p* < 0.01; SIN+SB‐505124 group versus SIN group ^&^ *p* < 0.05, ^&&^ *p* < 0.01, *n* = 12. (D–G) Double immunofluorescence staining exhibited the expression of Iba‐1 (green) with p‐Smad3 (red) in the ipsilateral spinal dorsal horn of sham and CCI mice after SIN treatment and intrathecal delivery of inhibitors (scale bar = 100 μm). (H–J) WB analysis showed the TGF‐β1, Iba‐1 and p‐Smad3 expression levels in the ipsilateral spinal dorsal horn of sham and CCI mice after SIN treatment and inhibitor intrathecal delivery. Data are presented as the mean ± SEM, sham group versus CCI group ***p < 0.001; SIN group versus CCI group ^#^ *p* < 0.05, ^###^ *p* < 0.001; SIN+SB‐505124 group versus SIN group ^&^ *p* < 0.05, ^&&^ *p* < 0.01, ^&&&^ *p* < 0.001, *n* = 6.

As previously reported, ALK1 and ALK5 are binding receptors for TGF‐β1 [32, 33]. Among them, ALK1 phosphorylates Smad1/5/8 and ALK5 phosphorylates Smad2/3, each leading to nuclear translocation of distinct molecular complexes producing different changes in gene expression [34]. The SIN‐treated CCI mice were intrathecally administered an ALK1 selective inhibitor (ML347) or ALK5 selective inhibitor (SB‐505124) to identify the receptor that initiates the TGF‐β1‐mediated intracellular signalling cascade of antimicroglial activation. Here, inhibition of spinal ALK5 reversed the rising trends of PWT and TWL in SIN‐treated CCI mice (PWT: repeated measure ANOVA *F*_4,55_ = 117.004, *p* < 0.001; TWL: repeated measure ANOVA *F*_4,55_ = 43.527, *p* < 0.001) (Figure 6B,C). The SIN induced Iba‐1 downregulation and p‐Smad3 upregulation in the ipsilateral spinal dorsal horn of CCI mice were also blocked by SB‐505124, despite the SIN evoking the release of TGF‐β1 at a high level (Iba‐1 positive area: one‐way ANOVA *F*_4,25_ = 13.330, *p* < 0.001; p‐Smad3 positive area: one‐way ANOVA *F*_4,25_ = 46.205, *p* < 0.001; colocalised area: one‐way ANOVA *F*_4,25_ = 25.975, *p* < 0.001; TGF‐β1/GAPDH: one‐way ANOVA *F*_4,25_ = 86.899, *p* < 0.001; Iba‐1/GAPDH: one‐way ANOVA *F*_4,25_ = 67.970, *p* < 0.001; p‐Smad3/GAPDH: one‐way ANOVA *F*_4,25_ = 188.960, *p* < 0.001) (Figure 6D–J). Therefore, blocking ALK5 at the spinal cord level abrogated the effects of SIN on pain sensitisation, microglial activation and Smad3 phosphorylation. Moreover, these results indicated that ALK5 might be the specific binding receptor to determine the modulative effect of TGF‐β1 on microglial activity.

### 3.5 Smad3 Modulated the TGFβ1‐Induced Microglial Inhibition

**FIGURE 7:**
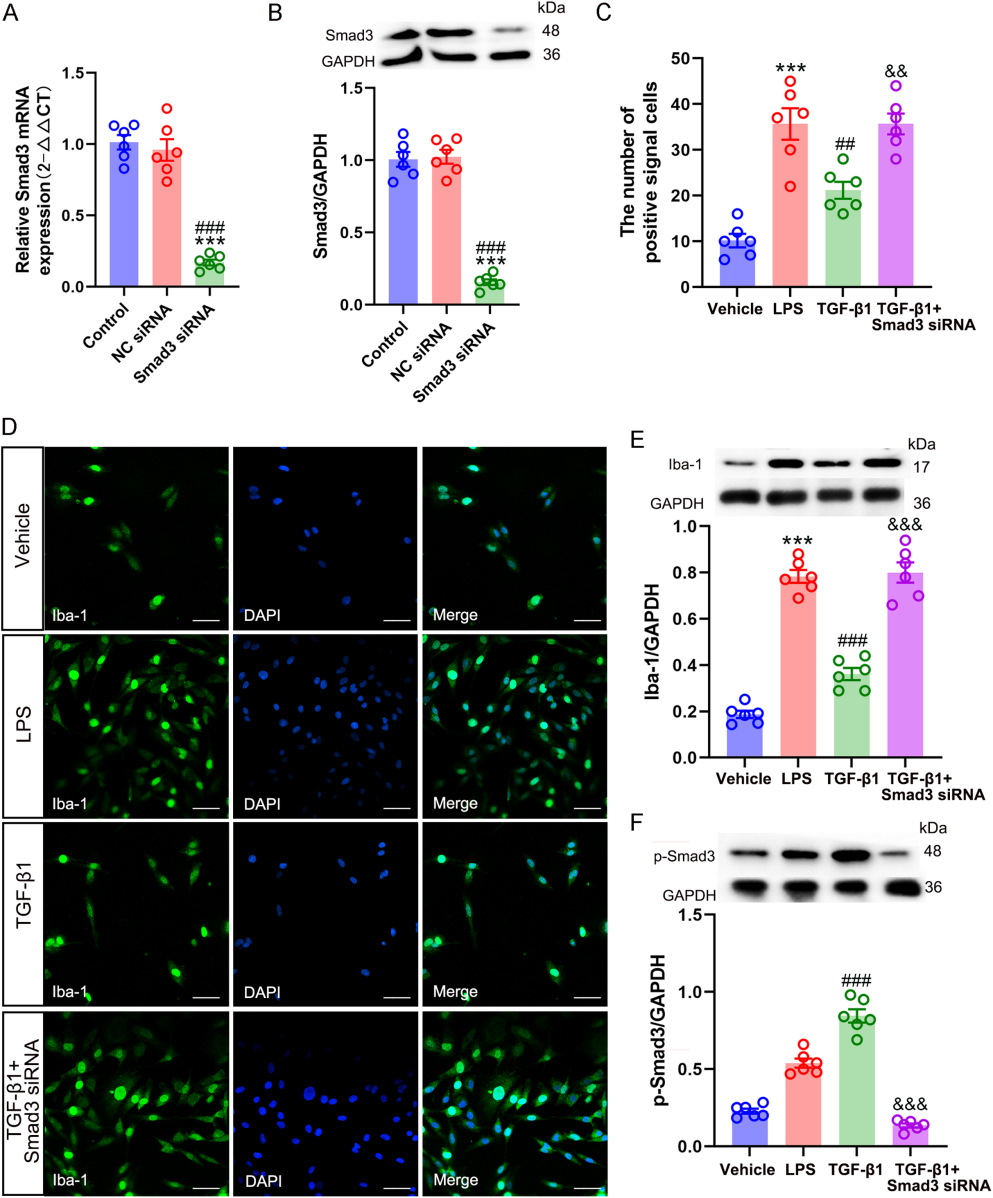
Smad3 is pivotal for the inhibitory effects of TGF‐β1 on LPS‐induced microglial activation. (A) qRT‐PCR analysis of Smad3 mRNA expression in cultured BV2 cells transfected with siRNAs. (B) WB analysis confirmed Smad3 protein expression in cultured BV2 cells transfected with siRNAs, data are presented as mean ± SEM, Smad3 siRNA group versus control group, ***p < 0.001; Smad3 siRNA group versus NC siRNA group, ^###^ *p* < 0.001, *n* = 6. (C and D) Immunofluorescence staining showed the Iba‐1 expression in cultured BV2 cells (scale bar = 50 μm). (E and F) WB analysis revealed Iba‐1 and p‐Smad3 expression in cultured BV2 cells. Data are presented as the mean ± SEM, vehicle group versus LPS group, ***p < 0.001; LPS group versus TGF‐β1 group, ^##^ *p* < 0.01, ^###^ *p* < 0.001; TGF‐β1 group versus TGF‐β1 + Smad3 siRNA group, ^&&^ *p* < 0.01, ^&&&^ *p* < 0.001, *n* = 6.

Having confirmed the involvement of TGFβ1/ALK5 in the SIN‐induced microglial inhibition, whether its downstream mediator Smad3 participated in this process needs further investigation in vitro. After 24 h of transfection (cell viability > 95%, 1 × 10^5^/mL), Smad3 mRNA and protein expression were significantly decreased in the Smad3 siRNA group compared to the NC siRNA and control groups (Smad3 mRNA: one‐way ANOVA *F*_2,15_ = 76.103, *p* < 0.001; Smad3/GAPDH: one‐way ANOVA *F*_2,15_ = 139.742, *p* < 0.001) (Figure 7A,B), indicating that Smad3 siRNA successfully decreased Smad3 expression in vitro. As described previously, LPS is a potent stimulator of cultured microglia [35]. Therefore, in vitro LPS treatment was used to mimic the CCI‐evoked microglial activation in the spinal dorsal horn. Following treatment with 100 ng/mL LPS for 12 h, Iba‐1 expression and Iba‐1 positive cell numbers were significantly increased, indicating the activation of BV2 microglial cells (Iba‐1 positive signal cell numbers: one‐way ANOVA *F*_3,20_ = 27.075, *p* < 0.001; Iba‐1/GAPDH: one‐way ANOVA *F*_3,20_ = 103.994, *p* < 0.001) (Figure 7C–E). As expected, 10 ng/mL of exogenetic TGFβ1 peptide vividly dampened BV2 cell activation and evoked Smad3 phosphorylation (p‐Smad3/GAPDH: one‐way ANOVA *F*_3,20_ = 55.679, *p* < 0.001) (Figure 7F). However, the exogenetic TGFβ1 peptide could not reverse the stimulatory effect of LPS on Smad3 siRNA transfected‐BV2 cells. These results indicated a key role of Smad3 in TGFβ1‐induced microglial inhibition.

### 3.6 Blocking of Smad3 Reversed SIN‐Induced Pain Alleviation and Microglial Inhibition in CCI Mice

**FIGURE 8:**
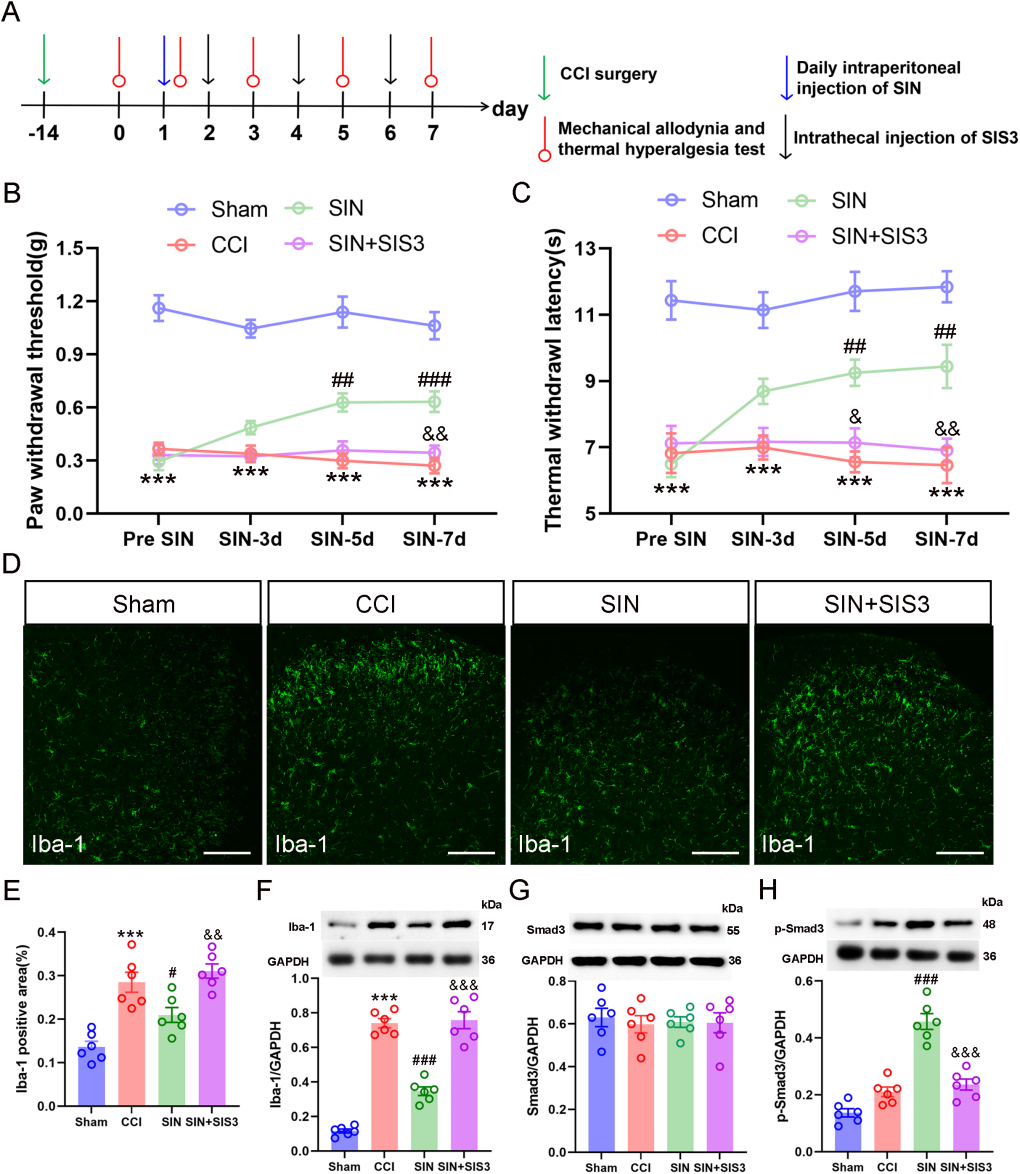
Intrathecal injection of a Smad3 inhibitor abrogated SIN‐induced pain amelioration and microglial inhibition. (A) Schematic for determining the involvement of Smad3 in the effects of the SIN. (B and C) Time course of the PWT and TWL in sham and CCI mice after SIN treatment and SIS3 intrathecal delivery, data are presented as the mean ± SEM, sham group versus CCI group ***p < 0.001; SIN group versus CCI group ^##^ *p* < 0.01, ^###^ *p* < 0.001; SIN+SIS3 group versus SIN group ^&^ *p* < 0.05, ^&&^ *p* < 0.01, *n* = 12. (D and E) Immunofluorescence staining of Iba‐1 in the ipsilateral spinal dorsal horn of sham and CCI mice after SIN treatment and SIS3 intrathecal delivery (scale bar = 100 μm). (F–H) WB analysis revealed Iba‐1, Smad3, and p‐Smad3 expression levels in the ipsilateral spinal dorsal horn of sham and CCI mice after SIN treatment and SIS3 intrathecal delivery. Data are presented as the mean ± SEM. Sham group versus CCI group ***p < 0.001; SIN group versus CCI group ^#^ *p* < 0.05, ^###^ *p* < 0.001; SIN+SIS3 group versus SIN group ^&&^ *p* < 0.01, ^&&&^ *p* < 0.001, *n* = 6.

Consistent with the in vitro study, we also observed that Smad3 plays a key role in the action of SIN in CCI mice via intrathecal delivery of 100 ng of Smad3 inhibitor (SIS3). As expected, SIS3 suppressed SIN‐induced Smad3 phosphorylation, while neither SIS3 nor SIN affected Smad3 expression (Smad3/GAPDH: one‐way ANOVA *F*_3,20_ = 0.12, *p* = 0.947; p‐Smad3/GAPDH: one‐way ANOVA *F*_3,20_ = 52.931, *p* < 0.001) (Figure 8G,H). In behavioural tests, inhibition of Smad3 phosphorylation by SIS3 potently restrained the upward trend of PWT and TWL SIN‐treated CCI mice (PWT: repeated measure ANOVA *F*_3,44_ = 114.771, *p* < 0.001; TWL: repeated measure ANOVA *F*_3,44_ = 49.628, *p* < 0.001) (Figure 8B,C). Additionally, SIS3 abrogated the inhibitory effect of SIN on Iba‐1 expression in the ipsilateral spinal dorsal horn of CCI mice (Iba‐1 positive area: one‐way ANOVA *F*_3,20_ = 19.596, *p* < 0.001; Iba‐1/GAPDH: one‐way ANOVA *F*_3,20_ = 101.061, *p* < 0.001) (Figure 8D–F). These results confirmed that Smad3 acts as an indispensable intracellular modulator of SIN‐induced pain alleviation and microglial inhibition.

## 4 Discussion

Increasing evidence has demonstrated that SIN exhibited analgesic effects in various chronic pain, especially neuropathic pain. However, the potential mechanisms remain unknown. In this study, we confirmed that SIN ameliorated pain hypersensitivity in CCI mice, probably by restraining the neuroinflammation induced by microglia, which were activated in the spinal dorsal horn of mice. Additionally, SIN significantly enhanced TGF‐β1 expression and Smad3 phosphorylation in a dose‐dependent manner. Knockdown of spinal TGF‐β1 reversed SIN‐induced amelioration of pain hypersensitivity and microglial activation in CCI mice. Intrathecal administration of an ALK5 inhibitor abrogated the beneficial effects of SIN. Furthermore, targeting Smad3 in vitro using siRNA potently reversed the inhibition of TGF‐β1 on microglial activation. Finally, antagonising Smad3 abrogated the SIN‐induced inhibition of microglial activation in CCI mice. These results suggest that the TGF‐β1/ALK5/Smad3 axis plays a key role in the antinociceptive effects of SIN in CCI mice, indicating its suppressive ability in microglia.

The SIN, an alkaloid originating from the root of the climbing plant, has been reported to have various biological activities [36, 37]. In clinical trials, SIN has been demonstrated to exhibit anti‐inflammatory and antinociceptive effects in patients with chronic inflammatory pain like rheumatoid arthritis [38]. In rodents, repeated administration of SIN could alleviate neuroinflammation in the dorsal root ganglia and mechanical allodynia in rats with spinal nerve ligation [39]. In the present study, we also found that SIN dose dependently alleviated the mechanical allodynia and thermal hyperalgesia in male mice with CCI. Following peripheral nerve injury, the central terminals of injured afferents promptly release several substances, including chemokines or ATPs, subsequently activating microglia in the spinal dorsal horn [40, 41]. Activated microglia release numerous inflammatory cytokines, contributing to the central sensitisation of pain transmission [42]. Therefore, aberrant microglial activation in the spinal dorsal horn is one of the critical causes of central sensitisation in neuropathic pain. As expected, the CCI‐induced overactivity of microglia and over‐release of inflammatory cytokines were suppressed by SIN. Previous studies have shown that SIN is not only an efficient pain killer but also a potent inhibitor of microglial activation for male and female rodents in several neuroinflammatory states [15, 16, 43]. It is known that the pain perception of females is more sensitive and fluctuant than that of males due to the differences in oestrous cycle and sex hormones [44, 45]. Therefore, we chose male mice as the subjects of the present study. Although the analgesic effects of SIN on CCI‐related neuropathic pain were only observed in male mice, both existing evidence and our findings suggest that the inhibition of microglial activation may be the core mechanism by which SIN treats neuropathic pain in both sexes.

TGF‐β1 is a potent anti‐inflammatory cytokine that exhibits protective effects on several neuropathological conditions, including neuropathic pain [46, 47]. In neuropathic pain, TGF‐β1 could suppress the development of neuropathic allodynia and attenuate CCI‐induced spinal neuroinflammation and microglial activation [48]. In the present study, the repetitive administration of SIN induced a dramatic upregulation of spinal TGF‐β1, consistent with its pain alleviation and microglial inhibition. In addition, the upregulated TGF‐β1 was observed to be primarily located in neurons and microglia. The secretion of TGF‐β was found to be enhanced in rodents treated with SIN and involved in SIN's anti‐inflammatory effect [49, 50]. As an important subtype of TGF‐β, thus TGF‐β1 might also be a critical mediator of SIN in the treatment of neuropathic pain. As expected, the knockdown of the spinal expression of TGF‐β1 by its siRNA completely reversed SIN's beneficial effects, including pain sensitisation and microglial activation. These results confirm the crucial role of TGF‐β1 in SIN‐induced antinociceptive action.

The action of TGF‐β1 requires the response of downstream signalling mediators, including TGF‐β type I and II receptors and Smad proteins [51, 52]. ALK1 and ALK5 are specific type I TGF‐β1 receptors. The SIN‐treated CCI mice were intrathecally administered an ALK1 selective inhibitor (ML347) or ALK5 selective inhibitor (SB‐505124) to distinguish which ALK receptors were involved in the TGF‐β1‐related antimicroglial effect. Blocking ALK5 at the spinal cord level abrogated SIN‐induced microglial activation and pain hypersensitivity, although the SIN evoked the release of TGF‐β1 at a high level. These results confirmed that ALK5 determines the role of TGF‐β1 in SIN‐induced microglial inhibition and antinociception.

Smad3 is a transcription factor and signal transduction molecule. Functionally, phosphorylated‐Smad3 couples with Smad‐binding elements in the promoter region to regulate gene expression [53]. Consistent with the upregulation of TGF‐β1, SIN also facilitated the phosphorylation of Smad3. In rodents, TGF‐β1/Smad3 signalling has been demonstrated to be a protective regulator against inflammatory transition [54]. After observing the upregulation of p‐Smad3 in the spinal cords following SIN treatment, we found that Smad3 is indispensable for TGF‐β1 to inhibit the activation of cultured BV2 microglial cells by transfection with Smad3 siRNA. The role of Smad3 in SIN effects was determined in vivo by intrathecal administration of a Smad3 inhibitor to SIN‐treated CCI mice. As expected, the Smad3 inhibitor restrained the beneficial effects of SIN on pain and spinal microglia in CCI mice. Based on the in vitro and in vivo results, Smad3 is well established as an intracellular mediator of SIN in modulating microglial activation.

In summary, this study discovered that SIN may attenuate CCI‐induced neuropathic pain by relieving spinal microglial activation via TGF‐β1 overexpression, thereby initiating Smad3 phosphorylation via ALK5. These results provide novel evidence supporting the clinical use of SIN for neuropathic pain treatment.

## Author Contributions

**Ling Ling:** data curation (equal), methodology (equal), resources (lead). **Min Luo:** methodology (equal), data curation (lead), investigation (lead). **Haolin Yin:** methodology (equal), software (equal). **Yunyun Tian:** methodology (equal). **Tao Wang:** methodology (equal). **Bangjian Zhang:** conceptualization (equal), formal analysis (equal). **Li Yin:** funding acquisition (equal), writing – original draft (supporting), writing – review and editing (supporting). **Yuehui Zhang:** formal analysis (lead), methodology (equal), validation (lead). **Jiang Bian:** conceptualization (equal), funding acquisition (equal), project administration (lead), writing – original draft (lead), writing – review and editing (lead).

## Ethics Statement

The authors have nothing to report.

## Conflicts of Interest

The authors declare no conflicts of interest.

## Data Availability

The data that support the findings of this study are available on request from the corresponding author. The data are not publicly available due to privacy or ethical restrictions.
